# Rapid Evaluation of Coronavirus Illness Severity (RECOILS) in intensive care: Development and validation of a prognostic tool for in‐hospital mortality

**DOI:** 10.1111/aas.13991

**Published:** 2021-10-15

**Authors:** Drago Plečko, Nicolas Bennett, Johan Mårtensson, Tariq A. Dam, Robert Entjes, Thijs C. D. Rettig, Dave A. Dongelmans, Age D. Boelens, Sander Rigter, Stefaan H. A. Hendriks, Remko de Jong, Marlijn J. A. Kamps, Marco Peters, Attila Karakus, Diederik Gommers, Dharmanand Ramnarain, Evert‐Jan Wils, Sefanja Achterberg, Ralph Nowitzky, Walter van den Tempel, Cornelis P. C. de Jager, Fleur G. C. A. Nooteboom, Evelien Oostdijk, Peter Koetsier, Alexander D. Cornet, Auke C. Reidinga, Wouter de Ruijter, Rob J. Bosman, Tim Frenzel, Louise C. Urlings‐Strop, Paul de Jong, Ellen G.M. Smit, Olaf L. Cremer, D. Jannet Mehagnoul‐Schipper, Harald J. Faber, Judith Lens, Gert B. Brunnekreef, Barbara Festen‐Spanjer, Tom Dormans, Daan P. de Bruin, Robbert C. A. Lalisang, Sebastiaan J. J. Vonk, Martin E. Haan, Lucas M. Fleuren, Patrick J. Thoral, Paul W. G. Elbers, Rinaldo Bellomo

**Affiliations:** ^1^ Department of Intensive Care Medicine Laboratory for Critical Care Computational Intelligence Amsterdam Medical Data Science Amsterdam UMC Amsterdam The Netherlands; ^2^ Department of Mathematics Seminar for Statistics ETH Zürich Zurich Switzerland; ^3^ Department of Physiology and Pharmacology Section of Anaesthesia and Intensive Care Karolinska Institutet Stockholm Sweden; ^4^ Department of Perioperative Medicine and Intensive Care Karolinska University Hospital Stockholm Sweden; ^5^ Department of Intensive Care Admiraal De Ruyter Ziekenhuis Goes The Netherlands; ^6^ Department of Intensive Care Amphia Ziekenhuis Breda The Netherlands; ^7^ Antonius Ziekenhuis Sneek Sneek The Netherlands; ^8^ Department of Anesthesiology and Intensive Care St. Antonius Hospital Nieuwegein The Netherlands; ^9^ Intensive Care Albert Schweitzerziekenhuis Dordrecht The Netherlands; ^10^ Intensive Care Bovenij Ziekenhuis Amsterdam The Netherlands; ^11^ Intensive Care Catharina Ziekenhuis Eindhoven Eindhoven The Netherlands; ^12^ Intensive Care Canisius Wilhelmina Ziekenhuis Nijmegen The Netherlands; ^13^ Department of Intensive Care Diakonessenhuis Hospital Utrecht The Netherlands; ^14^ Department of Intensive Care Erasmus Medical Center Rotterdam The Netherlands; ^15^ Intensive Care ETZ Tilburg Tilburg The Netherlands; ^16^ Department of Intensive Care Franciscus Gasthuis & Vlietland Rotterdam The Netherlands; ^17^ ICU Haaglanden Medisch Centrum Den Haag The Netherlands; ^18^ Intensive Care HagaZiekenhuis Den Haag The Netherlands; ^19^ Department of Intensive Care Ikazia Ziekenhuis Rotterdam Rotterdam The Netherlands; ^20^ Department of Intensive Care Jeroen Bosch Ziekenhuis Den Bosch The Netherlands; ^21^ Intensive Care Laurentius Ziekenhuis Roermond The Netherlands; ^22^ ICU Maasstad Ziekenhuis Rotterdam Rotterdam The Netherlands; ^23^ Intensive Care Medisch Centrum Leeuwarden Leeuwarden The Netherlands; ^24^ Department of Intensive Care Medisch Spectrum Twente Enschede The Netherlands; ^25^ ICU SEH, BWC Martiniziekenhuis Groningen The Netherlands; ^26^ Department of Intensive Care Medicine Northwest Clinics Alkmaar The Netherlands; ^27^ ICU OLVG Amsterdam The Netherlands; ^28^ Department of Intensive Care Medicine Radboud University Medical Center Nijmegen The Netherlands; ^29^ Intensive Care Reinier de Graaf Gasthuis Delft The Netherlands; ^30^ Department of Anesthesia and Intensive Care Slingeland Ziekenhuis Doetinchem The Netherlands; ^31^ Intensive Care Spaarne Gasthuis Haarlem en Hoofddorp The Netherlands; ^32^ Intensive Care UMC Utrecht Utrecht The Netherlands; ^33^ Intensive Care VieCuri Medisch Centrum Venlo The Netherlands; ^34^ ICU WZA Assen The Netherlands; ^35^ ICU ICU, IJsselland Ziekenhuis Capelle aan den IJssel The Netherlands; ^36^ Department of Intensive Care Ziekenhuisgroep Twente Almelo The Netherlands; ^37^ Intensive Care Ziekenhuis Gelderse Vallei Ede The Netherlands; ^38^ Intensive care Zuyderland MC Heerlen The Netherlands; ^39^ Pacmed, Amsterdam Amsterdam The Netherlands; ^40^ Australian and New Zealand Intensive Care Research Centre School of Public Health and Preventative Medicine Monash University Melbourne Australia; ^41^ Department of Critical Care The University of Melbourne Melbourne Australia; ^42^ Data Analytics Research and Evaluation Centre Department of Medicine and Radiology The University of Melbourne; ^43^ Austin Hospital Melbourne Australia

**Keywords:** corona virus, COVID‐19, intensive care, mechanical ventilation, respiratory failure

## Abstract

**Background:**

The prediction of in‐hospital mortality for ICU patients with COVID‐19 is fundamental to treatment and resource allocation. The main purpose was to develop an easily implemented score for such prediction.

**Methods:**

This was an observational, multicenter, development, and validation study on a national critical care dataset of COVID‐19 patients. A systematic literature review was performed to determine variables possibly important for COVID‐19 mortality prediction. Using a logistic multivariable model with a LASSO penalty, we developed the Rapid Evaluation of Coronavirus Illness Severity (RECOILS) score and compared its performance against published scores.

**Results:**

Our development (validation) cohort consisted of 1480 (937) adult patients from 14 (11) Dutch ICUs admitted between March 2020 and April 2021. Median age was 65 (65) years, 31% (26%) died in hospital, 74% (72%) were males, average length of ICU stay was 7.83 (10.25) days and average length of hospital stay was 15.90 (19.92) days. Age, platelets, PaO2/FiO2 ratio, pH, blood urea nitrogen, temperature, PaCO2, Glasgow Coma Scale (GCS) score measured within +/−24 h of ICU admission were used to develop the score. The AUROC of RECOILS score was 0.75 (CI 0.71–0.78) which was higher than that of any previously reported predictive scores (0.68 [CI 0.64–0.71], 0.61 [CI 0.58–0.66], 0.67 [CI 0.63–0.70], 0.70 [CI 0.67–0.74] for ISARIC 4C Mortality Score, SOFA, SAPS‐III, and age, respectively).

**Conclusions:**

Using a large dataset from multiple Dutch ICUs, we developed a predictive score for mortality of COVID‐19 patients admitted to ICU, which outperformed other predictive scores reported so far.

**Editorial Comment**

Using a large dataset from multiple Dutch ICUs, the authors developed a predictive score for mortality of COVID‐19 patients admitted to ICU, which outperformed other predictive scores reported so far.

## 1 INTRODUCTION

By 1st of April 2021, 129 million infections with severe acute respiratory coronavirus 2 (SARS‐CoV‐2) had been confirmed worldwide. At the same time, the resulting coronavirus disease (COVID‐19) had caused an estimated 2.8 million deaths.^1^, ^2^ Once patients are admitted to the Intensive Care Unit (ICU), COVID‐19 has a high mortality rate.^3^ Moreover, the large numbers of patients requiring hospitalization^4^ and/or ICU admission have put healthcare systems under immense pressure, with shortcomings in the availability and quality of many aspects of medical treatment.^5^, ^6^, ^7^

The severe form of COVID‐19 is most notably characterized by respiratory failure.^8^, ^9^, ^10^, ^11^, ^12^, ^13^, ^14^, ^15^, ^16^, ^17^, ^18^, ^19^, ^20^, ^21^ In these patients, predicting outcome in the first 24 h of ICU admission is fundamental to the safe, effective and appropriate allocation of key components of ICU treatment. In this regard, some demographic features and markers of illness severity have been reported as helpful in identifying patients at particularly high mortality risk. They have included older age,^10^, ^12^, ^13^, ^14^, ^15^, ^16^, ^17^, ^19^, ^20^, ^22^, ^23^, ^24^, ^25^, ^26^, ^27^, ^28^, ^29^, ^30^ male sex,^11^, ^12^, ^14^, ^24^, ^25^, ^31^ various comorbidities,^11^, ^13^, ^14^, ^15^, ^17^, ^18^, ^25^, ^31^, ^32^, ^34^ acute kidney injury,^21^, ^33^, ^35^ coagulation problems,^10^, ^13^, ^17^, ^22^, ^27^, ^30^, ^36^, ^37^ increased markers of inflammation,^12^, ^13^, ^15^, ^26^, ^28^, ^29^, ^30^, ^34^ abnormal blood cell counts^12^, ^19^, ^23^, ^28^, ^34^, ^37^, ^38^ and, in one study, increased hepatobiliary markers.^21^ The prognostic accuracy of individual markers, however, is limited, which led to the development of more complex multivariate predictive scores.

Several risk scores have been constructed with the intention of predicting outcome of patients infected with SARS‐CoV‐2, most importantly mortality.^12^, ^14^ The benefit of good quality predictive scores is twofold. First, they can have direct clinical utility if used to stratify patients based on risk, which is often required for triage purposes. Second, they can provide a useful tool in clinical research, where the need to adjust randomization for illness severity is key.

To the best of our knowledge, however, there are still no large, multicenter studies comparing different clinical risk scores for predicting mortality among ICU patients with COVID‐19. In this development and validation study, our aim was to take advantage of a large multicenter national database from the Netherlands to construct a novel risk score based on such more detailed and granular data and to study its performance compared to that of previously published scores. In particular, we aimed to test the hypothesis that a better performing predictive score could be developed using routinely collected ICU data to predict in‐hospital mortality in COVID‐19 patients admitted to ICU.

## 2 METHODS

### 2.1 Study design and cohort

This was a retrospective, multicenter, observational study in which we developed and validated a prognostic score for the primary outcome of in‐hospital mortality. The study cohort consisted of adults (>18 years) admitted to intensive care units (ICUs) with a confirmed SARS‐CoV‐2 infection, between March 2020 and April 2021, across 25 different hospitals in the Netherlands. Patients who were still in the hospital at the time of writing of this manuscript were excluded from the analysis, together with patients who were transferred to other hospitals, which were not part of the Dutch COVID‐19 database. Patients who were discharged from hospital, but were readmitted at a later stage, were treated as separate patient encounters.

A systematic literature review was conducted, in order to determine all the currently reported risk factors for COVID‐19 mortality. A flowchart documenting the literature review process is shown in Figure S1 and a comprehensive list of variables that were found during this search is given in Table S1. The variables were broadly categorized into the following groups: age^10^, ^12^, ^13^, ^14^, ^15^, ^16^, ^17^, ^19^, ^20^, ^30^ and comorbidities,^11^, ^13^, ^14^, ^15^, ^17^, ^18^, ^23^, ^24^, ^25^, ^31^, ^32^, ^33^, ^34^ respiratory,^8^, ^9^, ^10^, ^11^, ^12^, ^13^, ^14^, ^15^, ^16^, ^17^, ^18^, ^19^, ^20^, ^21^ renal,^21^, ^33^, ^35^ coagulation,^10^, ^13^, ^17^, ^22^, ^27^, ^30^, ^36^, ^37^ inflammatory,^12^, ^13^, ^15^, ^26^, ^28^, ^29^, ^30^, ^34^ metabolic,^17^, ^19^, ^29^, ^30^, ^34^, ^36^, ^37^, ^38^ cell counts,^12^, ^19^, ^23^, ^28^, ^34^, ^37^, ^38^ central nervous system.^11^, ^14^, ^31^

We then determined the worst recorded value of each of the variables in the 48‐h period around ICU admission (ICU admission +/−24 h). After this, the area under receiver operator characteristic curve (AUROC) was inspected for each candidate feature found in the literature. Additionally, a tree ensemble (Random Forest) model was constructed for predicting mortality, with emphasis on inspecting feature importance (based on the Gini index) within such a tree‐based model. This resulted in a selection of 10 variables that were most predictive. Patients who had three or more missing variables were excluded from the cohort. The remaining missing values were imputed, assuming missingness was a sign that a variable lied in the clinically normal range.

The study cohort was split into an approximately 60% development set and 40% validation set. The splitting was based on hospitals, so that model validation data originated from hospitals which were not included in the development cohort. The size of the splits was chosen in order to have more than 855 patients in the validation cohort, which ensured a power of more than 75% of detecting an AUROC improvement of 0.05 over a baseline score with AUROC 0.7, for a test with a 5% significance level, assuming independent predictors.

Instead of selecting variable cut‐off points manually by inspecting ROC curves, we determined a clinically relevant range for a feature value, together with an increase that would be deemed clinically significant. For example, the relevant range for blood urea nitrogen was taken to be between 15 and 100 mg/dl, where an increase of 5 mg/dl was deemed significant. The selection of relevant thresholds and associated weights was left to the logistic regression model with a least absolute shrinkage and selection operator (LASSO) penalty.

Several clinical risk scores were considered for comparison with our newly developed score, including the Sequential Organ Failure Assessment (SOFA) score, the Simplified Acute Physiology Score (SAPS‐III), and the International Severe Acute Respiratory and Emerging Infection Consortium (ISARIC) 4C mortality score. A baseline using only age as a continuous predictor was also included.

The performance of the predictive scores was evaluated by inspecting the receiver operator characteristic (ROC) and precision‐recall (PR) curves, together with the commonly used measure of area under the curve (AUC). The PR curve indicates the positive predictive value (PPV) of a score, for each value of sensitivity. We reported the 95% confidence intervals (CIs) of points on the ROC and PR curves, together with confidence intervals of AUROC and area under PR curve (AUPRC), which were obtained using bootstrap. We tested several null hypotheses H_0_ in order to determine whether our score significantly improved performance upon the above predictive scores. Due to a smaller proportion of women in the cohort, in a sensitivity analysis, we inspected the ROC curve when splitting the entire cohort on sex. Additionally, we also inspected the ROC curve when splitting the cohort based on admission wave (first wave defined as admissions before August 2020, second wave admissions after August 2020). Lastly, we inspected the calibration of our newly developed score, reporting the average mortality rate (with a 95% CI) for each score level. Also, as the score was derived based on a logistic model, we provided a formula to compute the expected mortality rate based on the score value.

The Medical Ethics Committee at Amsterdam UMC, location VUmc waived the need for patient informed consent and approved of an opt‐out procedure for the collection of COVID‐19 patient data during the COVID‐19 crisis. For statistical analysis and data loading, we used the ricu R‐package^39^ and R Statistical Software^40^ Version 4.0.0. All the code used in the analyses is available on Github https://github.com/eth‐mds/recoils.

## 3 RESULTS

The database consisted of 2901 adult patients who were discharged from or died in hospital. Due to missingness of key variables, 484 patients were excluded from the study, yielding a final study cohort of 2417 patients. In development and validation cohorts, respectively, the median age was 65 and 65 years, 31% and 26% of patients died in hospital, 74% and 72% of patients were male, the average length of ICU stay was 7.83 and 10.25 days and the average length of hospital stay was 15.90 and 19.92 days. Detailed information on patient demographics in the development and validation cohorts is provided in Table 1, in which we note that the mortality rate was lower in the validation cohort.

**TABLE 1:** Patient characteristics and outcomes

Variable	Reported	Development cohort	Validation cohort
Cohort size	*n*	1480	937
Age (years)	Median (IQR)	65 (57–72)	65 (57–72)
Mortality	%	31	26
ICU LOS	Median (IQR)	7.83 (3.48–14.75)	10.25 (3.89–19.35)
Hospital LOS (days)	Median (IQR)	15.9 (9.5–26.86)	19.92 (11.75–34.31)
Gender (Female)	%	26	28
Gender (Male)	%	74	72
Ventilated patients	*n* (%)	1108 (75)	685 (73)
Acute Kidney Injury	*n* (%)	79 (5)	83 (9)
Chronic respiratory insufficiency	*n* (%)	51 (3)	51 (5)
Diabetes	*n* (%)	264 (18)	140 (15)
Chronic dialysis	*n* (%)	2 (0)	2 (0)
Chronic renal insufficiency	*n* (%)	44 (3)	30 (3)
COPD	*n* (%)	103 (7)	65 (7)
PaO2/FiO2 (mmHg)	Median (IQR)	86.95 (65.26–135.74)	82.94 (63–129.13)
Urea (mg/dl)	Median (IQR)	22.12 (15.12–32.2)	21.56 (15.12–33.32)
C‐reactive protein (mg/L)	Median (IQR)	148.5 (82.75–234.25)	157 (95–242)
Glasgow coma scale	Median (IQR)	15 (7–15)	15 (7–15)
Respiratory rate (insp/min)	Median (IQR)	33 (28–40)	36 (30–44)
PaCO2 (mmHg)	Median (IQR)	45 (38.25–53)	45 (37.5–54)
Temperature (C)	Median (IQR)	38.22 (37.4–39.16)	38.2 (37.39–39.1)
Platelets (10^9^/L)	Median (IQR)	232 (174.75–298)	231 (166–301)
Arterial blood pH	Median (IQR)	7.36 (7.29–7.42)	7.35 (7.27–7.42)

Comorbidities reported in the table follow the definitions of the National Institute for Health and Care Excellence (NICE).

Abbreviations: C, degrees Celsius; COPD, chronic obstructive pulmonary disease; LOS, length of stay; mmHg, millimeters of mercury.

The AUROC and importance in a tree ensemble model for every feature included in the analysis are given in Table S2, together with the clinically relevant range and clinically significant increase/decrease values. Based on these values, the variables that were selected for score construction included: age, C‐reactive protein, platelets, PaO2/FiO2 ratio, arterial blood pH, blood urea nitrogen, temperature, PaCO2, respiratory rate, and Glasgow Coma Scale (GCS) score. The median and interquartile ranges for these variables can be found in Table 1.

We evaluated a total of 4773 CRP, 5184 platelets, 28 090 PaO2/FiO2, 14 416 arterial blood pH, 4681 urea nitrogen, 32 248 temperature, 14 441 arterial CO2 partial pressure, 49 790 respiratory rate, and 7609 Glasgow Coma Scale measurements collected in the 48‐h period around ICU admission.

**FIGURE 1:**
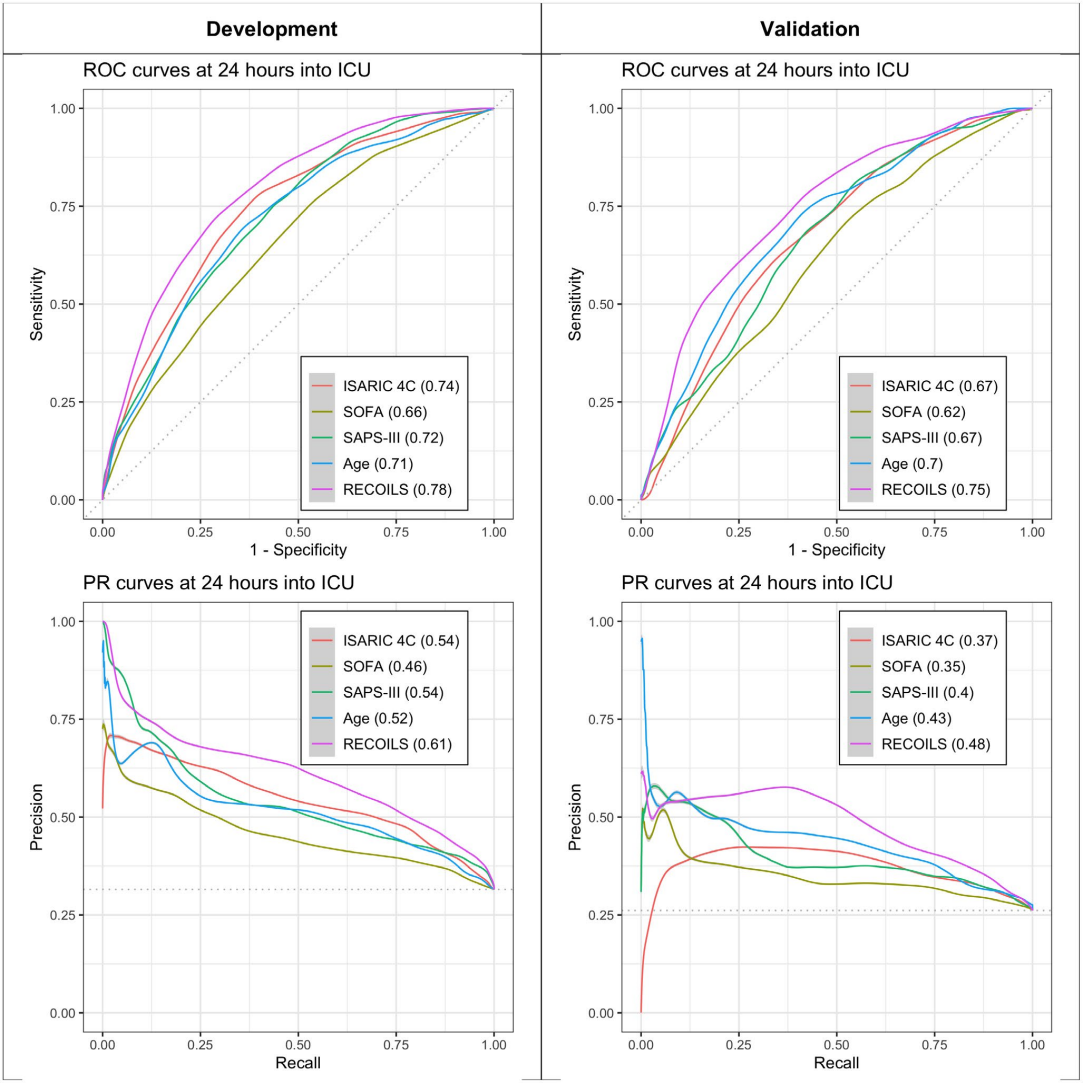
Evaluation of RECOILS score on development and validation cohorts. ROC and PR curves of the Rapid Evaluation of Coronavirus Illness Severity (RECOILS) in the development and validation cohorts are presented, together with various possible baseline methods. The AUROC and AUPRC values are shown in brackets next to each score, respectively

The development split consisted of 1480 patients from 14 hospitals and the validation split of 937 patients from 11 hospitals. The score derived from the LASSO logistic model selected eight prediction variables and was named Rapid Evaluation of Coronavirus Illness Severity (RECOILS) and is presented in Table 2. The β‐coefficient estimates from which the score was constructed are presented in Table S3. The ROC and PR curves of the score, alongside clinical baseline scores, are presented in Figure 1. Splitting the cohort based on sex (*p* = .32) and admission wave (*p* = .46) did not yield significantly different ROC curves (Figure S2).

**TABLE 2:** Rapid Evaluation of Coronavirus Illness Severity (RECOILS)

	Points
Age (years)	
<60	—
≥60	2
≥65	4
≥70	6
≥75	7
PaCO2 (mmHg)	
<60	—
≥60	1
≥72	3
Glasgow coma scale (points)	
≥7	—
<7	1
PaO2/FiO2 (mmHg)	
≥300	—
<300	1
<100	2
Arterial blood pH	
≥7.4	—
<7.4	1
<7.3	2
<7.15	3
<6.85	6
Platelets (10^9/L)	
≥120	—
<120	1
<40	3
Temperature (C)	
<38	—
≥38	1
Urea nitrogen (mg/dl)	
<30	—
≥30	1
≥35	2
≥40	3

Values of every subcomponent are added together to obtain the final score.

**FIGURE 2:**
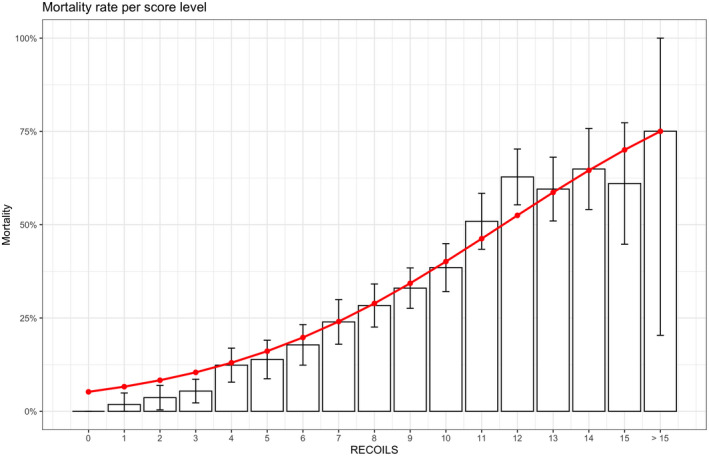
Calibration of RECOILS score. Increasing values of the RECOILS score are associated with increased mortality, showing good score calibration. The average mortality rate for different values of the RECOILS score, with 95% confidence intervals are shown as bars. The red line represents the mortality risk estimated based on the formula provided in the main text

The RECOILS score achieved an AUROC of 0.75 (CI 0.71–0.78) and AUPRC of 0.48 (CI 0.41–0.54) in the validation cohort and was the score with the best performance in both development and validation cohorts (Table 3). The null hypotheses that, for the validation cohort, the AUROC of RECOILS was indistinguishable from that of other published risk scores, were all rejected at a 5% significance level. Thus, the AUROC of RECOILS was greater than all other scores published so far (AUROC values 0.68 [CI 0.64–0.71], 0.61 [CI 0.58–0.66], 0.67 [CI 0.63–0.70], 0.70 [CI 0.67–0.74] for ISARIC 4C Mortality Score, SOFA, SAPS‐III, and age, respectively). Model calibration was inspected by plotting the mortality rate for each value of the score (Figure 2) and the model was found to be well calibrated (Brier score 0.167). The equation that can be used to estimate the expected mortality rate based on the RECOILS score is given by

$$p\approx \frac{e^{\left(‐2.9+\frac{\text{RECOILS}}{4}\right)}}{1+e^{\left(‐2.9+\frac{\text{RECOILS}}{4}\right)}}$$

**TABLE 3:** Performance of clinical risk scores in predicting in‐hospital mortality of COVID‐19 patients admitted to ICU

Score	AUROC (95% CI) development	AUPRC (95% CI) development	AUROC (95% CI) validation	AUPRC (95% CI) validation
ISARIC 4C	0.74 (0.714–0.766)	0.543 (0.496–0.591)	0.675 (0.638–0.712)	0.371 (0.32–0.422)
SOFA	0.655 (0.626–0.684)	0.457 (0.413–0.501)	0.615 (0.576–0.655)	0.347 (0.294–0.4)
SAPS‐III	0.723 (0.696–0.749)	0.539 (0.493–0.585)	0.669 (0.632–0.706)	0.398 (0.338–0.459)
Age	0.713 (0.685–0.740)	0.516 (0.469–0.564)	0.703 (0.666–0.74)	0.434 (0.373–0.498)
RECOILS	0.784 (0.761–0.808)	0.609 (0.561–0.657)	0.745 (0.709–0.781)	0.478 (0.416–0.541)

Values of AUROC and AUPRC, with 95% confidence intervals, are shown for development and validation cohorts.

The comparison of the observed mortality rates for each value of RECOILS with the estimates based on the above formula is reported graphically in Figure 2 and also in Table S4.

## 4 DISCUSSION

### 4.1 Key findings

Using a large national database, we developed a novel risk score for predicting in‐hospital mortality for critically ill COVID‐19 patients, based on systematically selected variables and a data‐driven choice of important variable thresholds. The score was developed using data from 14 different ICUs and was validated on a separate set of ICUs, making the findings more robust and generalizable. The score was significantly superior to previously reported competing clinical scores both in terms of AUROC and AUPRC. Lastly, we provided a simple formula that calculates the expected mortality proportion based on the score value.

### 4.2 Relationship to previous literature

All the variables utilized in our predictive score have appeared in previous literature on COVID‐19 and mortality, with the possible exception of arterial blood pH. Our findings confirm previous literature, which identified COVID‐19 mortality as associated with older age, respiratory acidosis, acute kidney injury, low platelets, and damage to the central nervous system. However, by identifying important thresholds and combining variables into a multivariate score, we significantly improved predictive performance over existing scores.

### 4.3 Implications of study findings

Our findings imply that it is possible to construct a predictive score for mortality of COVID‐19 patients admitted to the ICU, which can be evaluated within 24 h of ICU admission, and that outperforms all published predictive scores so far. Moreover, our proposed score can be used for clinical research, to adjust any identified effects of treatment according to baseline risk and also to compare outcomes across different hospital centers based on the expected mortality proportion for each score value. Finally, this score can also be used in trials to stratify randomization according to baseline risk.

### 4.4 Strengths and limitations

There are several strengths to our study. We conducted a multicenter study, involving 25 different hospitals and almost 2500 patients, making it the largest study on clinical prediction scores for ICU patients with COVID‐19 so far. The systematic review of the variables reported in the literature and the data‐driven approach to threshold selection served to minimize bias in the score construction. Additionally, the validation of the score on a set of hospitals, separate from those in the development cohort, makes the findings of this study more likely to be both robust and generalizable.

We also acknowledge several limitations to our study. It is an observational study, therefore prone to possible sampling bias, and causal inferences cannot be drawn from this study. Second, even though the Dutch COVID‐19 database contains information on most of the important comorbidities identified to be associated with mortality from COVID‐19, it is possible that some comorbidities were underreported. Third, some previously published risk scores, such as the ISARIC 4C mortality score, were designed for the emergency room setting. For this reason, they suffer in performance when applied to the ICU setting. This, however, emphasizes the importance of developing a risk score specific to the ICU setting. Lastly, the Dutch intensive care system is that of a resource‐rich country and the findings of our predictive score are likely to be relevant to similar systems, but less likely to be directly relevant to ICU systems in middle or low‐income countries.

## 5 CONCLUSION

In a multicenter study involving over 2400 COVID‐19 ICU patients admitted to 25 different hospitals across the Netherlands, we developed and externally validated a predictive risk score (RECOILS) for in‐hospital mortality, which significantly outperformed all COVID‐19 specific outcome prediction scores published thus far. This score can be used by clinicians to help prognosticate; make decisions in relation to resource allocation; assist in treatment efficacy assessment; and help stratify patients for randomization in clinical trials.

## CONFLICTS OF INTEREST

The authors report no conflicts of interest.

## AUTHOR CONTRIBUTION

RB contributed important clinical concepts and study design. DP designed and conducted the statistical analysis. DP, JM, and RB wrote the manuscript. DP and NB developed the supporting computational infrastructure. NB revised the manuscript. TD, RE, TR, DD, AB, SR, SH, RJ, MK, MP, AK, DG, DR, EJW, SA, RN, WT, CJ, FN, EO, PK, AC, AR, RB, TF, LUS, PD, ES, OC, JMS, HF, JL, GB, BFS, TD, DB, RL, SV, MH, LF, PT, and PE contributed to the collection and construction of the Dutch COVID‐19 data warehouse.

## CODE AVAILABILITY

All code available on https://github.com/eth‐mds/recoils.

## ETHICS APPROVAL

The Medical Ethics Committee at Amsterdam UMC, location VUmc waived the need for patient informed consent and approved of an opt‐out procedure for the collection of COVID‐19 patient data during the COVID‐19 crisis.

## CONSENT TO PARTICIPATE

Not applicable.

## CONSENT FOR PUBLICATION

All authors consent to the publication of the manuscript.

## Supporting information

Fig S1‐S2

Table S1‐S4

## Data Availability

The data used are not publicly available.
