# Supplementary material for: Rapid Evaluation of Coronavirus Illness Severity (RECOILS) in intensive care: Development and validation of a prognostic tool for in‐hospital mortality
Source: Acta Anaesthesiol Scand. 2021 Oct 15;66(1):65–75. doi: 10.1111/aas.13991 (PMC8652966; doi:10.1111/aas.13991)

**Supplementary Figure 1**. Literature review flowchart.

A flowchart indicating the steps of the systematic literature review that was conducted, including the date of the investigation and the filter that was used for the search.

**
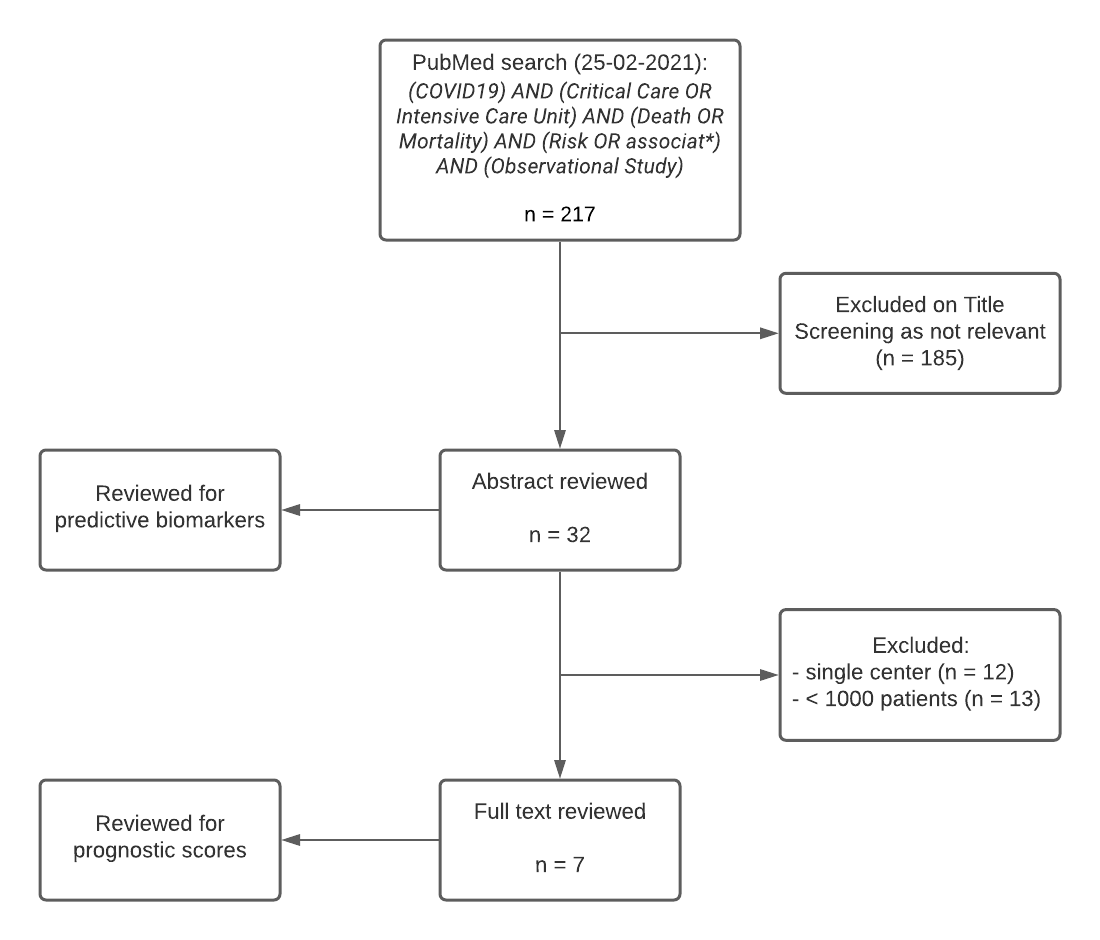
**

**Supplementary Figure 2**. RECOILS score for different sex and admission waves.

ROC curves of the RECOILS score remain stable when the cohort was split based on sex (p = 0.32) or admission wave (p = 0.46).


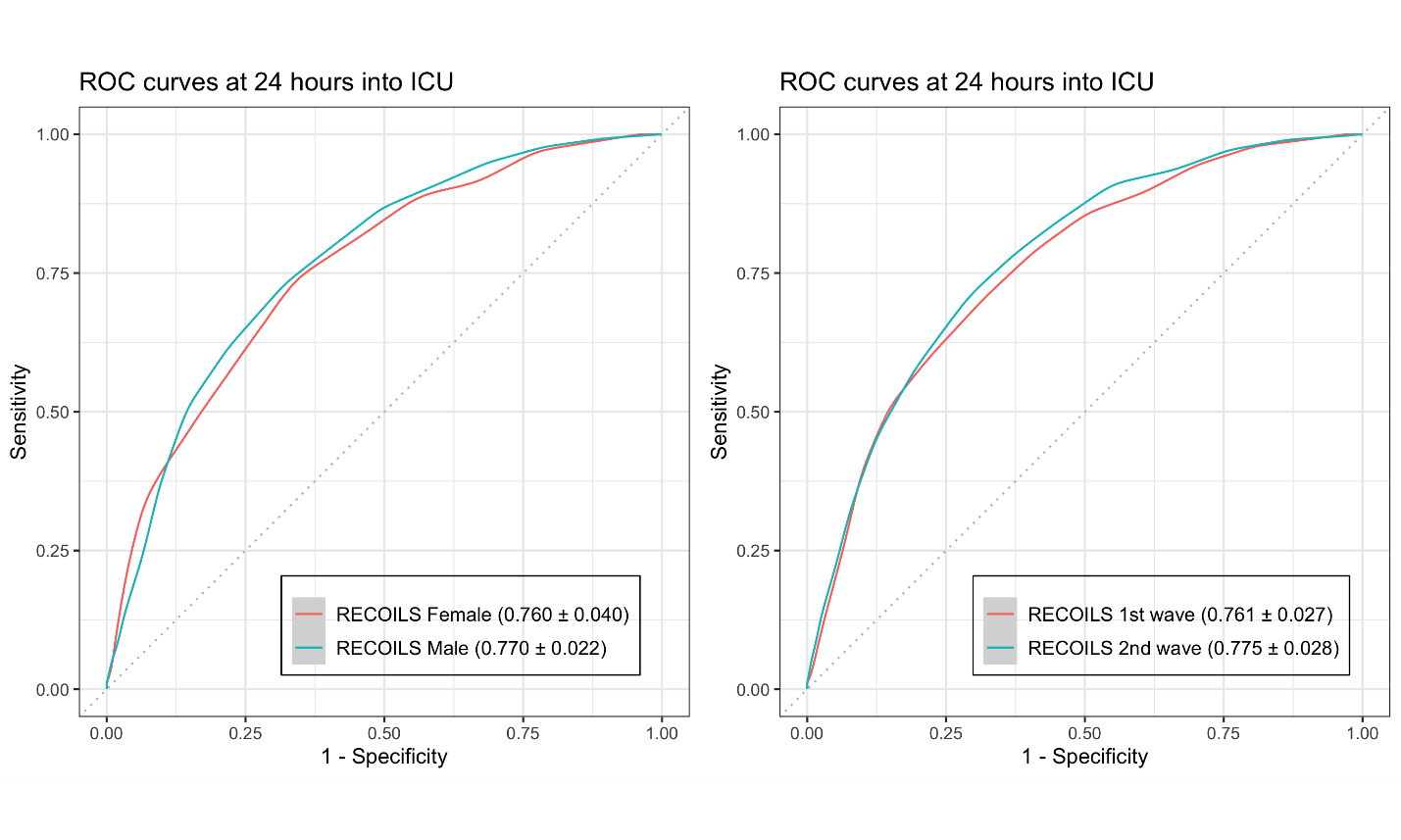

Supplement: Supplementary file 1 — Fig S1‐S2 [file AAS-66-65-s002.docx]
