# Supplementary material for: Rapid Evaluation of Coronavirus Illness Severity (RECOILS) in intensive care: Development and validation of a prognostic tool for in‐hospital mortality
Source: Acta Anaesthesiol Scand. 2021 Oct 15;66(1):65–75. doi: 10.1111/aas.13991 (PMC8652966; doi:10.1111/aas.13991)
Supplement: Supplementary file 2 — Table S1‐S4 [file AAS-66-65-s001.docx]

**Supplementary Table 1.** Variables found to be associated with COVID-19 mortality in previous literature.

| Category | Variables |
| --- | --- |
| Age | Age |
| Metabolic | Lactate, Bicarbonate, Arterial Blood pH, Lactate-Dehydrogenase |
| Coagulation | D-Dimer, Platelets, INR(PT), APTT, Fibrinogen |
| Inflammation | Procalcitonin, C-Reactive Protein, Temperature |
| Respiratory | Lung Compliance, Ventilatory Ratio, PaO2/FiO2, SaO2/FiO2, PaCO2 - EtCO2, PaO2, PaCO2, Oxygen Saturation, Respiratory Rate |
| Cell Counts | Lymphocytes, Neutrophils, Neutrophil/Lymphocyte Ratio, Basophils, Eosinophils, White Blood Cell Count |
| Comorbidities | Comorbidity Score* |
| Liver | Total Bilirubin, Alkaline Phosphatase, Phosphate, Albumin |
| Renal | Chloride, Creatinine, Magnesium, Potassium, Sodium, Urea Nitrogen |
| Central Nervous System | Glasgow Coma Scale score |

*Comorbidity Score is calculated as the number of comorbidities a patient has, among those reported in the database: acute renal failure, chronic dialysis, chronic renal insufficiency, cirrhosis, chronic obstructive pulmonary disease, diabetes, hematologic malignancy, immunodeficiency, neoplasm, chronic respiratory insufficiency, vascular insufficiency.

**Supplementary Table 2.** AUROC, feature importance in a tree ensemble model (using Gini index), clinically relevant range, increase or decrease and imputation value for every feature used in the preliminary analysis.

| Feature / marker | AUROC | Feature importance | Relevant range | Significant increase/decrease | Imputation value |
| --- | --- | --- | --- | --- | --- |
| Age (years) | 0.702 | 122.22 | 25-80 | 5 | 65 |
| Arterial blood pH | 0.629 | 42.09 | 6.5-7.4 | -0.05 | 7.42 |
| Creatinine (mg/dL) | 0.616 | 45.64 | 1-14 | 1 | 0.8 |
| Urea nitrogen (mg/dL) | 0.593 | 38.69 | 15-50 | 5 | 10 |
| CO2 partial pressure (mmHg) | 0.592 | 38.11 | 46-86 | 2 | 40 |
| PaCO2 - EtCO2 (mmHg) | 0.582 | 25.69 | 0-100 | 10 | 0 |
| Lung compliance (mL/cmH_2_0) | 0.580 | 41.85 | 0-200 | -10 | 200 |
| Potassium (mEq/L) | 0.579 | 28.52 | 4-8.4 | 0.2 | 3.8 |
| Platelets (10^9^/L) | 0.576 | 38.55 | 0-200 | -20 | 250 |
| Ventilatory ratio | 0.563 | 23.04 | 2-15 | 1 | 1 |
| Lactate (mmol/L) | 0.562 | 29.07 | 1.01-10 | 0.5 | 1 |
| Bicarbonate (mmol/L) | 0.561 | 30.98 | 5-25 | -2 | 25 |
| SaO2/FiO2 (%) | 0.559 | 28.54 | 0-450 | -50 | 450 |
| Comorbidity score | 0.557 | 23.68 | 0-4 | 1 | 0 |
| PaO2/FiO2 (mmHg) | 0.553 | 33.96 | 70-99 | -1 | 99.5 |
| Oxygen saturation (%) | 0.548 | 22.62 | 3-14 | -1 | 15 |
| Glasgow coma scale (points) | 0.545 | 14.23 | 10-500 | 20 | 5 |
| C-reactive protein (mg/L) | 0.543 | 30.30 | 0-400 | -50 | 450 |
| Total bilirubin (mg/dL) | 0.535 | 24.40 | 1-30 | 1 | 0.8 |
| White blood cell count (K/uL) | 0.534 | 29.94 | 10-35 | 1 | 9 |
| Lactate-dehydrogenase (U/L) | 0.529 | 28.78 | 280-500 | 20 | 140 |
| Phosphate (mg/dL) | 0.527 | 27.63 | 3.4-12.4 | 0.5 | 2 |
| Temperature (C) | 0.524 | 45.10 | 37.5-40.5 | 0.25 | 36.8 |
| O2 partial pressure (mmHg) | 0.520 | 28.38 | 55-98 | -2 | 100 |
| INR(PT) | 0.520 | 13.49 | 1.1-3 | 0.2 | 1 |
| Albumin (g/dL) | 0.516 | 22.46 | 0-3.4 | -0.2 | 3.8 |
| Lymphocytes (%) | 0.514 | 16.30 | 0-20 | -1 | 25 |
| Respiratory rate (insp/min) | 0.510 | 40.05 | 14-36 | 2 | 12 |
| Alkaline phosphatase (IU/L) | 0.510 | 25.26 | 104-500 | 20 | 80 |
| APTT (sec) | 0.510 | 23.85 | 25-100 | 5 | 24 |
| Fibrinogen (g/L) | 0.509 | 12.44 | 300-1500 | 100 | 250 |
| Eosinophils (%) | 0.504 | 9.56 | 0-2 | -0.1 | 2 |
| Basophils (%) | 0.501 | 13.53 | 0-0.2 | -0.01 | 0.25 |
| Sodium (mEq/L) | 0.497 | 22.90 | 115-140 | -2 | 142 |
| D-dimer (mmol/L) | 0.495 | 15.05 | 500-2000 | 100 | 250 |
| Neutrophil/lymphocyte ratio | 0.487 | 12.89 | 2-25 | 1 | 1.5 |
| Neutrophils (%) | 0.486 | 13.31 | 40-90 | 5 | 2.5 |
| Magnesium (mg/dL) | 0.473 | 30.32 | 2-4.3 | 0.1 | 1.5 |
| Procalcitonin (ng/mL) | 0.468 | 18.06 | 0.15-20 | 0.1 | 0.05 |
| Chloride (mEq/L) | 0.466 | 21.37 | 80-104 | -2 | 104 |

**Supplementary Table 3.** Estimated β-coefficient values from which the score is constructed.

| Score threshold | β-coefficient | β-coefficient x4 (rounded) |
| --- | --- | --- |
| Age ≥ 50 years | 0.068 | 0 |
| Age ≥ 60 years | 0.511 | 2 |
| Age ≥ 65 years | 0.428 | 2 |
| Age ≥ 70 years | 0.558 | 2 |
| Age ≥ 75 years | 0.143 | 1 |
| PaCO2 ≥ 60 mmHg | 0.333 | 1 |
| PaCO2 ≥ 72 mmHg | 0.406 | 2 |
| C-reactive protein ≥ 110 mg/L | 0.036 | 0 |
| C-reactive protein ≥ 250 mg/L | 0.030 | 0 |
| C-reactive protein ≥ 390 mg/L | 0.005 | 0 |
| Glasgow coma scale < 7 points | 0.257 | 1 |
| PaO2/FiO2 < 300 mmHg | 0.197 | 1 |
| PaO2/FiO2 < 150 mmHg | 0.067 | 0 |
| PaO2/FiO2 < 100 mmHg | 0.259 | 1 |
| PaO2/FiO2 < 50 mmHg | 0.028 | 0 |
| Arterial blood pH < 7.4 | 0.189 | 1 |
| Arterial blood pH < 7.3 | 0.243 | 1 |
| Arterial blood pH < 7.15 | 0.321 | 1 |
| Arterial blood pH < 6.85 | 0.802 | 3 |
| Platelets < 200 * 10^9/L | 0.023 | 0 |
| Platelets < 120 * 10^9/L | 0.365 | 1 |
| Platelets < 100 * 10^9/L | 0.112 | 0 |
| Platelets < 60 * 10^9/L | 0.040 | 0 |
| Platelets < 40 * 10^9/L | 0.447 | 2 |
| Temperature ≥ 38 C | 0.225 | 1 |
| Temperature ≥ 38.25 C | 0.081 | 0 |
| Temperature ≥ 39 C | 0.045 | 0 |
| Urea nitrogen ≥ 30 mg/dL | 0.210 | 1 |
| Urea nitrogen ≥ 35 mg/dL | 0.137 | 1 |
| Urea nitrogen ≥ 40 mg/dL | 0.295 | 1 |

**Supplementary Table 4.** Comparison of the observed mortality rates (with 95% confidence intervals) with mortality estimates based on the formula $p \approx\frac{e^{\left( -2.9 + \frac{RECOILS}{4} \right)}}{1+ e^{\left( -2.9 + \frac{RECOILS}{4} \right)}}$ .

| RECOILS score | Empirical estimate of mortality (%) (95% CI) | Model estimate of mortality (%) |
| --- | --- | --- |
| 0 | 0 (0-0) | 5 |
| 1 | 2 (0-6) | 7 |
| 2 | 4 (1-7) | 8 |
| 3 | 6 (2-9) | 10 |
| 4 | 12 (8-17) | 13 |
| 5 | 13 (9-18) | 16 |
| 6 | 17 (12-22) | 20 |
| 7 | 24 (18-30) | 24 |
| 8 | 27 (22-33) | 29 |
| 9 | 33 (27-39) | 34 |
| 10 | 38 (31-44) | 40 |
| 11 | 51 (44-58) | 46 |
| 12 | 63 (55-71) | 52 |
| 13 | 59 (51-68) | 59 |
| 14 | 65 (55-76) | 65 |
| 15 | 63 (48-78) | 70 |
| 16 | 68 (52-85) | 75 |
| 17 | 51 (24-79) | 79 |
| 18 | 59 (20-100) | 83 |
| 19 | 100 (100-100) | 86 |
| 20 | 100 (100-100) | 89 |
